# A Rare Anatomical Finding: Bilateral Accessory Mental Foramen

**DOI:** 10.1155/2021/6020515

**Published:** 2021-08-21

**Authors:** Zülfikar Karabıyık, Mustafa Kıranatlı

**Affiliations:** ^1^Kütahya Health Science University, Dentistry Faculty, Department of Oral and Maxillofacial Surgery, Turkey; ^2^Kütahya Health Science University, Dentistry Faculty, Department of Oral and Maxillofacial Radiology, Turkey

## Abstract

**Aim:**

Accessory mental foramen (AMF) is a not common anatomical variation. During the surgical procedures involving the mandible such as implant surgery, periapical surgery, jaw surgeries, and periapical surgery and enucleation of pathologies at the mental region, obvious attention should be given to prevent postoperative sequelae. *Case Report*. Orthopantomograph (OPG) is routinely taken to visualize the maxillofacial region at a dental clinic. OPG shows exactly upper and lower jaw and teeth but superficially reveals some pathology or anatomic variation. It misses sometimes an anatomic landmark such as AMF. As the surgery is planned to a maxillofacial region, a detailed knowledge should be known before going into surgery to not interfere with anatomic landmarks. A 52-year-old male patient was referred to Kütahya Health Science University Dental Hospital, Turkey, to rehabilitate his bilateral partial edentulous lower jaw region. Implant surgery was planned in our patient. OPG was taken to evaluate the maxillofacial region but was unremarkable. Before the implant surgery, CBCT was obtained from our patient. CBCT and a three-dimensional reconstructed model of the male patient showed bilateral accessory mental foramen (AMF).

**Conclusion:**

Accessory mental foramen (AMF) carries additional innervation to the chin, mandibular anterior gingiva, and mental region. Reflection and protection of the AMF during the surgery can prevent hemorrhage and neurosensory disturbance at the mental region and can improve quality of life for the patient. CBCT has higher precision but also a higher price and radiation dose. Although anatomical variations are uncommon, they can be found on digital panoramic radiographs but in limited percentage.

## 1. Introductıon

The path and shape of the mandibular canal and mental foramen are significant milestones in surgical operations performed on the mandible such as implant placement, periapical surgeries, enucleations of pathologies, surgical correction of jaw deformities, and extraction of impacted teeth [1]. The mental foramen (MF) is placed on the anterolateral side of the mandible [2, 3]. MF is generally located in an inferior position which is between root apexes of the bicuspids. It usually opens a superior-posterior position and carries neurovascular bundle named as the mental nerve. The mental nerve branches while it was exiting from the MF. The mental nerve innervates the skin of the mental and lower lip region. A buccal mucous membrane and buccal gingiva from the lower midline to the second premolar region is also innervated by the mental nerve [4].

Any foramen in addition to MF is regarded as accessory mental foramen (AMF) [2, 5]. Accessory foramen showing a connection with the mandibular canal is defined as accessory mental foramen, and accessory foramen revealing no connection with the mandibular canal is defined as nutrient foramen [6]. AMF is generally smaller than MF [7]. It can be found in the apical region of the first molar and in the posterior or superior region of the mental foramen as in our case. Detailed knowledge regarding the possible location, incidence, shape, and size of the AMF helps clinician to perform local anesthesia correctly and adequately. Identification of AMF on radiograph prevents misdiagnosis of bone lesions [8]. Identification and protection of AMF result in operation involving the mental region with minimal nerve injury and morbidity [9].

The present paper reports a case of AMF using CBCT.

## 2. A Case Report

**Figure 1:**
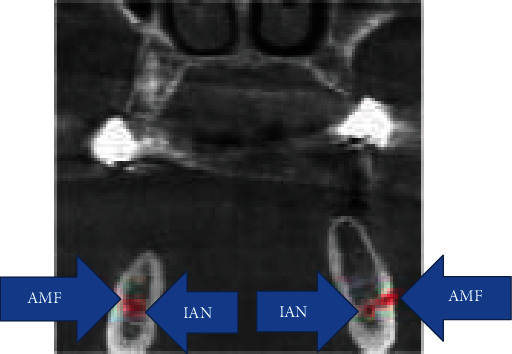
Right and left AMF at coronal CBCT.

A 52-year-old male patient came to the Department of Oral and Maxillofacial Surgery, Kütahya Health Science University Dental Hospital, Turkey, for the implant rehabilitation. His medical history was unremarkable. On the clinical examination, he was partially edentulous. On the panoramic radiograph, MF was identified. For the detailed examination, CBCT was obtained from the patient. Bilateral small round-shaped radiolucency distal to MF was seen at the axial CBCT section and a three-dimensional reconstructed model. These small foramina were connected to the inferior alveolar nerve canal (IAN) (Figure 1). It was referred to as AMF.

## 3. Discussion

**Figure 2:**
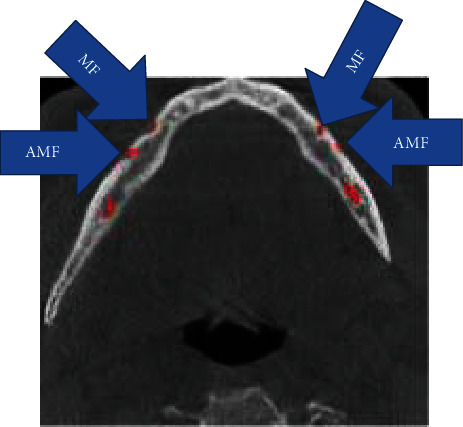
Right and left AMF at axial CBCT.

**Figure 3:**
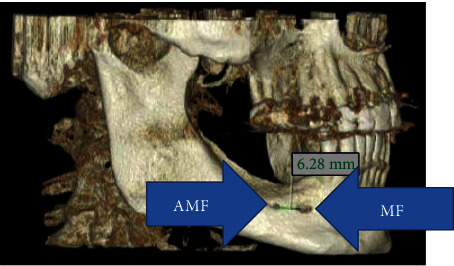
Right AMF at reconstructed CBCT.

**Figure 4:**
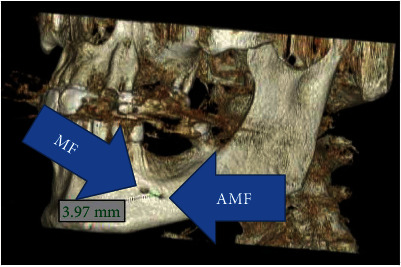
Left AMF at reconstructed CBCT.

**Figure 5:**
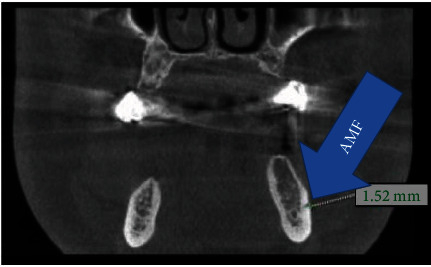
Left AMF at coronal CBCT.

**Figure 6:**
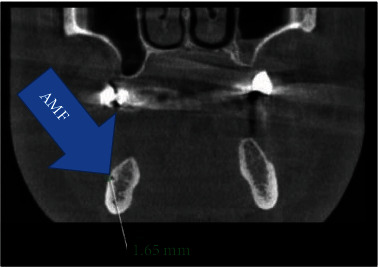
Right AMF at coronal CBCT.

**Figure 7:**
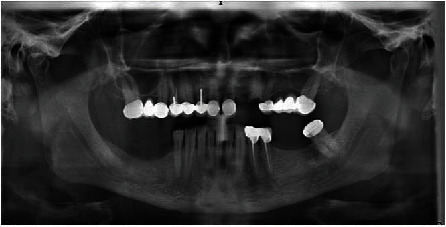
Patient's orthopantomograph.

MF is generally only one exit on each side of the lower jaw. MF is not developed until the 12th gestational week. Balcioğlu and Kocaelli asserted that splitting of the mental nerve from the inferior alveolar nerve before the exit to mental foramen may be a reason for the formation of AMF [2]. Double mental foramina are reported in literature [10]. AMF can change from a small indistinct bar to separate foramina [11]. Sawyer et al. added that the number of AMF can change from one to three foramina [4]. In the present case, two distinct foramina distal to MF were seen at axial CBCT and 3D reconstructed model bilaterally (Figures 2–4). Prevalence of AMF ranges from 1.4 to 12% [11–14]. Studies showed no gender differences according to the incidence of AMF [2]. Paraskevas et al. studied cadavers and found that the average diameter of AMF was 1 mm [8]. In our case, the diameter of the AMF was found to be 1.65 mm at the right side and 1.52 mm at the left site (Figures 5 and 6). AMF incidence can differ in ethnic groups [15]. Its incidence does not differ in right and left sides. Paraskevas et al. found that the mean distance from MF to AMF is 5.24 mm [8]. In our case, the distance from the MF to AMF was found to be 6.28 mm at the right side and 3.97 mm at the left side. MF can be observed by periapical radiography and OPG, but it can be detected with CBCT without exception [2]. AMF was detected on CBCT not on orthopantomograph in our case (Figure 7).

While it can be difficult to differentiate MF from AMF on radiographs, the presence of connection between the mandibular canal and such foramina supports the case of AMF.

## 4. Conclusıon

Neurosensory sequelae during the surgery involving the mental region are not scarce. In order to avoid neurovascular complications during the surgery, a possibility of AMF should be kept in mind. Knowledge about anatomic variations can prevent the complications and increase the quality of life after the surgery. CBCT has higher precision but also a higher price and radiation dose. Although anatomical variations (AMF) are uncommon, they can be found on digital panoramic radiographs but in limited percentage. AMF is detected on CBCT without exception.
